# Gamma Irradiation Does Not Induce Detectable Changes in DNA Methylation Directly following Exposure of Human Cells

**DOI:** 10.1371/journal.pone.0044858

**Published:** 2012-09-14

**Authors:** Christoph Lahtz, Steven E. Bates, Yong Jiang, Arthur X. Li, Xiwei Wu, Maria A. Hahn, Gerd P. Pfeifer

**Affiliations:** 1 Department of Cancer Biology, Beckman Research Institute, City of Hope, Duarte, California, United States of America; 2 Department of Information Sciences, City of Hope, Duarte, California, United States of America; 3 Department of Molecular Medicine, City of Hope, Duarte, California, United States of America; The University of Arizona, United States of America

## Abstract

Environmental chemicals and radiation have often been implicated in producing alterations of the epigenome thus potentially contributing to cancer and other diseases. Ionizing radiation, released during accidents at nuclear power plants or after atomic bomb explosions, is a potentially serious health threat for the exposed human population. This type of high-energy radiation causes DNA damage including single- and double-strand breaks and induces chromosomal rearrangements and mutations, but it is not known if ionizing radiation directly induces changes in the epigenome of irradiated cells. We treated normal human fibroblasts and normal human bronchial epithelial cells with different doses of γ-radiation emitted from a cesium 137 (^137^Cs) radiation source. After a seven-day recovery period, we analyzed global DNA methylation patterns in the irradiated and control cells using the methylated-CpG island recovery assay (MIRA) in combination with high-resolution microarrays. Bioinformatics analysis revealed only a small number of potential methylation changes with low fold-difference ratios in the irradiated cells. These minor methylation differences seen on the microarrays could not be verified by COBRA (combined bisulfite restriction analysis) or bisulfite sequencing of selected target loci. Our study shows that acute γ-radiation treatment of two types of human cells had no appreciable direct effect on DNA cytosine methylation patterns in exposed cells.

## Introduction

Three different types of radiation are released as a result of nuclear disintegration. Alpha (α) and beta (β) radiation consist of particles including the ionized and positively charged Helium core in case of α-radiation and an electron (positron) in case of β^−^ (β^+^)-radiation. In contrast to that, gamma (γ) radiation consists of electromagnetic waves with wavelength usually smaller than 0,005 nm; it is the radiation with the highest energy (≥200 keV) and penetration ability. All three kinds of radiation are able to ionize atoms or molecules through displacement of an electron (ionizing radiation). The major destructive effect of ionizing radiation in biological systems is based on radiolysis of water. The end product of this process is the hydroxyl radical (•OH). The hydroxyl radical can damage DNA and can introduce mutations [1], [2]. Radiation-induced DNA single- and double-strand breaks may be repaired erroneously leading to chromosomal rearrangements.

An important γ-radiation source in the present-day world is the artificial nuclide Cesium 137 (^137^Cs). Cesium 137 is a product of nuclear fission in nuclear power plants and is produced during atomic bomb explosions. The radioactive half-life of ^137^Cs is 30.17 years and it decays via β^−^ radiation with a likelihood of 93.5% indirectly to the metastable Barium 137 (^137m^Ba), which decays further with a half-life of 2.55 min via γ radiation into the stable nuclide Barium 137 (^137^Ba). Through above-ground atomic bomb tests a total of 948×10^15^ Bq of ^137^Cs have been released into the environment. Further, 85×10^15^ Bq of ^137^Cs were released through the “major accident level 7″ disaster in Chernobyl [3], and 35×10^15^ Bq were released in Fukushima Dai-ichi [4], [5]. From these sources combined, a total of 1,068×10^18^ Bq have been released during the past ∼60 years. Through radioactive fallout from these accidents and atomic tests, many areas have been contaminated. Because potassium is chemically similar to cesium, ^137^Cs becomes enriched in fungi, plants and animals and eventually enters into the human food chain.

**Figure 1:**
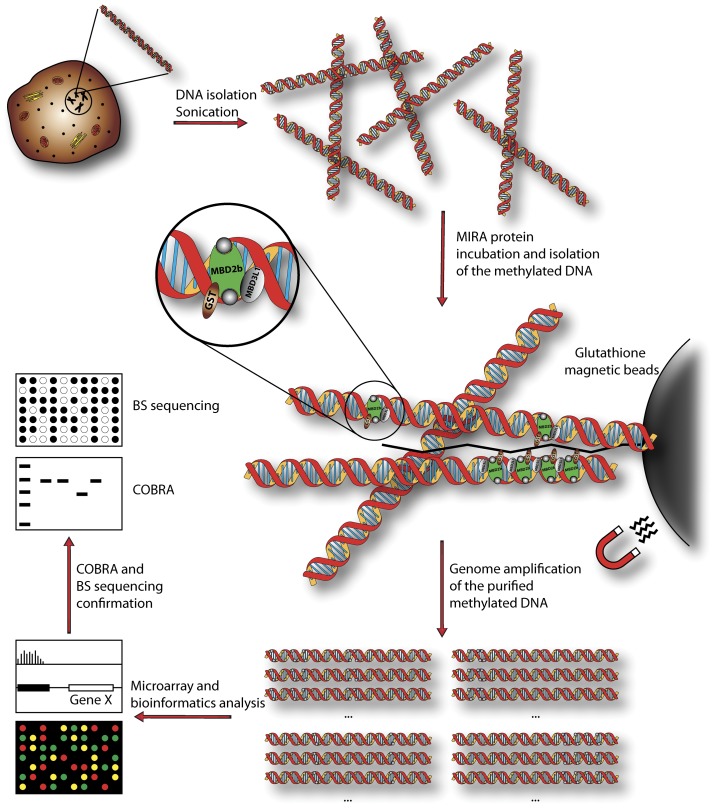
Schematic description of the MIRA method. The diagram shows the different steps of the procedure. After DNA isolation and sonication, the DNA fragments are incubated with the methyl-CpG binding protein complex (MBD2b and MBD3L1) and the bound methylated DNA is purified with glutathione-conjugated magnetic beads capturing the complex via GST-tagged MBD2b. After end repair and linker ligation (not shown in the picture), a genome amplification reaction is performed. The amplified DNA is hybridized to a microarray. After bioinformatics analysis, the detected hyper and/or hypomethylated peaks are confirmed with COBRA (combined bisulfite restriction analysis) and bisulfite sequencing.

**Table 1:** Number of peak differences in different comparisons.

Normalization	Quantile				Loess + Quantile			
Cutoff	Log2(2)		Log2(3)		Log2(2)		Log2(3)	
Comparison	Hyper	Hypo	Hyper	Hypo	Hyper	Hypo	Hyper	Hypo
HBEC: 0.1 Gy vs. 0 Gy, 7d	136	159	1	1	77	59	4	0
HBEC: 1 Gy vs. 0 Gy, 7d	36	126	1	3	60	73	0	3
HBEC: 4 Gy vs. 0 Gy, 7d	147	211	1	2	63	39	0	1
HBEC: 4 Gy, 7d vs. 4 Gy, 0d	218	224	1	12	67	17	0	0
HFB: 0.1 Gy vs. 0 Gy, 7d	3	9	0	0	7	3	0	0
HFB: 1 Gy vs. 0 Gy, 7d	60	118	3	0	9	7	1	0
HFB: 4 Gy vs. 0 Gy, 7d	8	54	0	0	5	6	0	0
HFB: 10 Gy vs. 0 Gy, 7d	3	6	0	0	11	4	0	0
HFB: 10 Gy, 7d vs. 10 Gy, 0d	27	46	0	0	70	27	0	0

**Figure 2:**
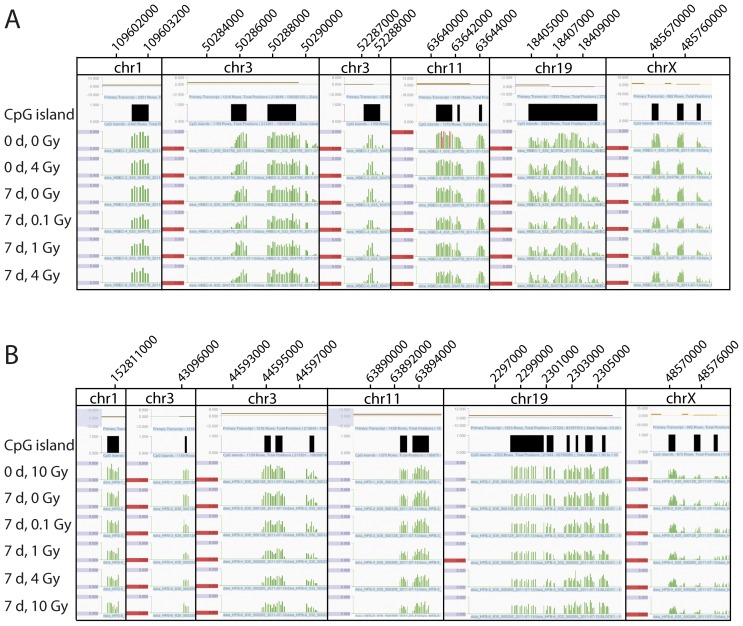
Examples of DNA methylation patterns in untreated and in irradiated cells. The Figure shows several randomly selected Signalmap snapshots of the microarray signals (green) for chromosomes 1, 3, 11, 19 and the X chromosome after the indicated treatments and recovery times. Above the treatment description are the CpG islands (indicated in black). **A.** Signalmap snapshots of HBEC (human bronchial epithelial cells) with the treatments 0 days, 0 Gray; 0 days, 4 Gray; 7 days, 0 Gray; 7 days, 0.1 Gray; 7 days, 1 Gray; 7 days, 4 Gray. **B.** Signalmap snapshots of HFB (human fibroblasts) with the treatments 0 days, 10 Gray; 7 days, 0 Gray; 7 days, 0.1 Gray; 7 days, 1 Gray; 7 days, 4 Gray; 7 days, 10 Gray.

The biological half-life of ^137^Cs in humans is 85 days for a 70 kg person [6], [7]. While ^137^Cs is incorporated, it damages tissue and cells, mainly through hydroxyl radicals. The dimension of the total influence of radioactive nuclides in biological systems is specified through the equivalent dose. The equivalent dose makes different types of radiation comparable. The equivalent dose is the result of the multiplication of the energy dose (Gray, Gy) with the relative biological effectiveness. In case of γ-radiation the equivalent dose in Sievert (Sv = 1 J/kg) is identical to the energy dose in Gray (1 Gy = 1 J/kg). Because of its DNA damaging ability, γ-radiation is used extensively in cancer therapy.

**Figure 3:**
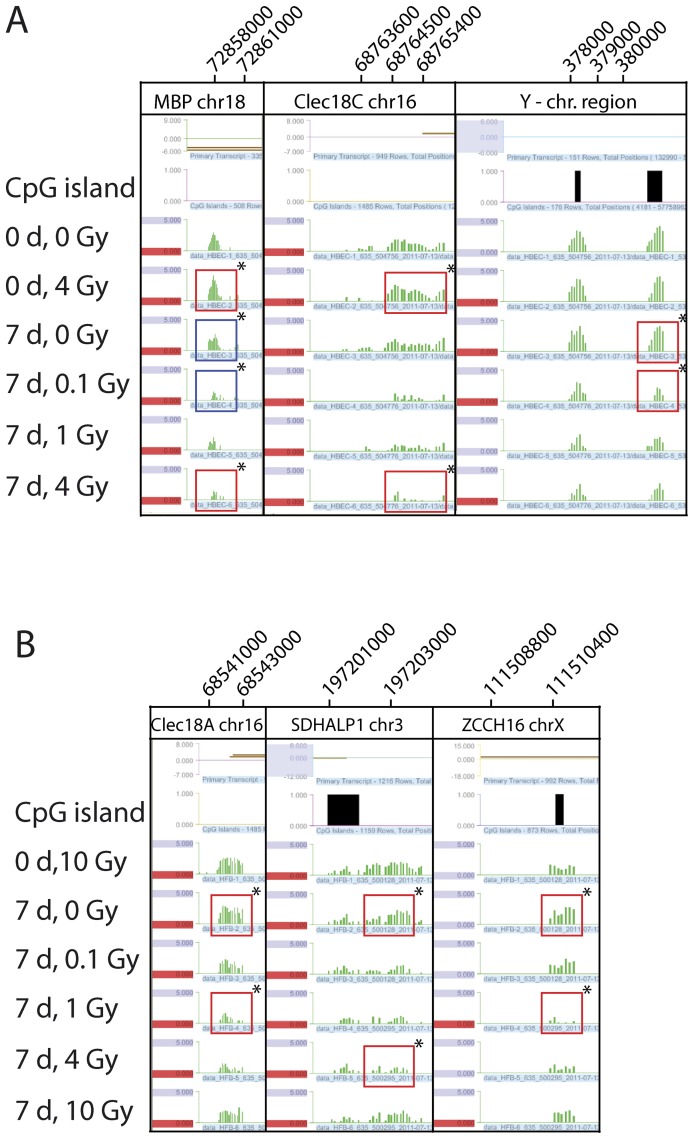
Apparently altered methylation patterns in certain genes. The picture shows six Signalmap snapshots of the microarray signals (green) from apparently differentially methylated genes after the particular radiation treatments and recovery times. Above the treatment description are the CpG islands (black). The signals framed by the red and blue rectangles are significantly (*) different methylated pairs of a control and a treatment, as determined by bioinformatics analysis. **A.** Signalmap snapshots of the genes *MBP* (0 days, 4 Gray vs. 7 days, 4 Gray (red rectangles) and 7 days, 0 Gray vs. 7 days, 0.1 Gray (blue rectangles), *CLEC18C* (0 days, 4 Gray vs. 7 days, 4 Gray (red rectangles) and a Y chromosomal region (7 days, 0 Gray vs. 7 days, 0.1 Gray (red rectangles) of HBEC. **B.** Signalmap snapshots of the genes *CLEC18A* (7 days, 0 Gray vs. 7 days, 1 Gray, red rectangles), *SDHALP1* (7 days, 0 Gray vs. 7 days, 4 Gray, red rectangles) and *ZCCHC16* (7 days, 0 Gray vs. 7 days 1 Gray, red rectangles) of HFB cells.

**Figure 4:**
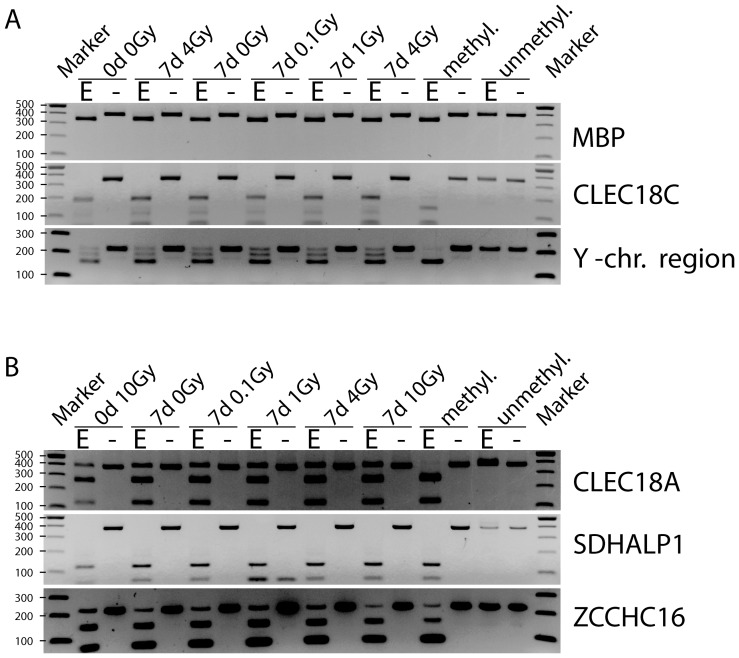
Methylation analysis of genes with suspected differentially methylated regions. DNA was treated with sodium bisulfite and amplified with conversion-specific COBRA (combined bisulfite restriction analysis) PCR primers. After the treatment with CpG specific restriction enzymes (E), these digests and a mock restriction digest (-) were run on a 2% agarose gel together with a 100 bp marker for analysis. A methylated (methyl.) and an unmethylated (unmethyl.) bisulfite-treated DNA sample were used as controls. **A.** COBRA gels of the genes *MBP*, *CLEC18C* and a Y chromosomal region in the HBEC cells with gamma radiation treatments 0 days, 0 Gray; 0 days, 4 Gray; 7 days, 0 Gray; 7 days, 0.1 Gray; 7 days, 1 Gray and 7 days, 4 Gray. **B.** COBRA gels of genes *CLEC18A*, *SDHALP1* and *ZCCHC16* in the HFB cells with the gamma radiation treatments 0 days, 10 Gray; 7 days, 0 Gray; 7 days, 0.1 Gray; 7 days, 1 Gray; 7 days, 4 Gray and 7 days, 10 Gray.

The DNA damaging effects of gamma radiation are well studied with thousands of publications in the literature investigating the mechanisms and biological outcomes of these effects. However, very little is known about any direct epigenetic effects that ionizing radiation may have in irradiated cells. Epigenetic regulatory mechanisms involve heritable marking of the DNA or histones; they are not associated with alterations of the DNA sequence. These epigenetic changes are generally reversible, but can be carried over with high fidelity to the daughter cells during DNA replication [8]. There are two main types of epigenetic mechanisms, one based on DNA cytosine methylation and the second based on histone modifications. These two types of epigenetic marks are often interconnected and can depend on each other. The cytosines at CpG dinucleotides in promoter regions of genes can be methylated often leading to long-term gene suppression [9], [10]. These DNA methylation events play a major role in epigenetic regulation in mammals [11], [12], [13]. Methylation patterns are often aberrant in cancer cells with global DNA hypomethylation and region-specific hypermethylation seen at CpG islands. The origin of aberrant DNA methylation in tumors is unknown. It has often been invoked that environmental exposures, for example chemicals or radiation, can initiate aberrant DNA methylation thus directly contributing to tumorigenesis. In this study, we have investigated if ionizing radiation from a ^137^Cs source can produce altered DNA methylation patterns in radiation-exposed primary human cells.

**Figure 5:**
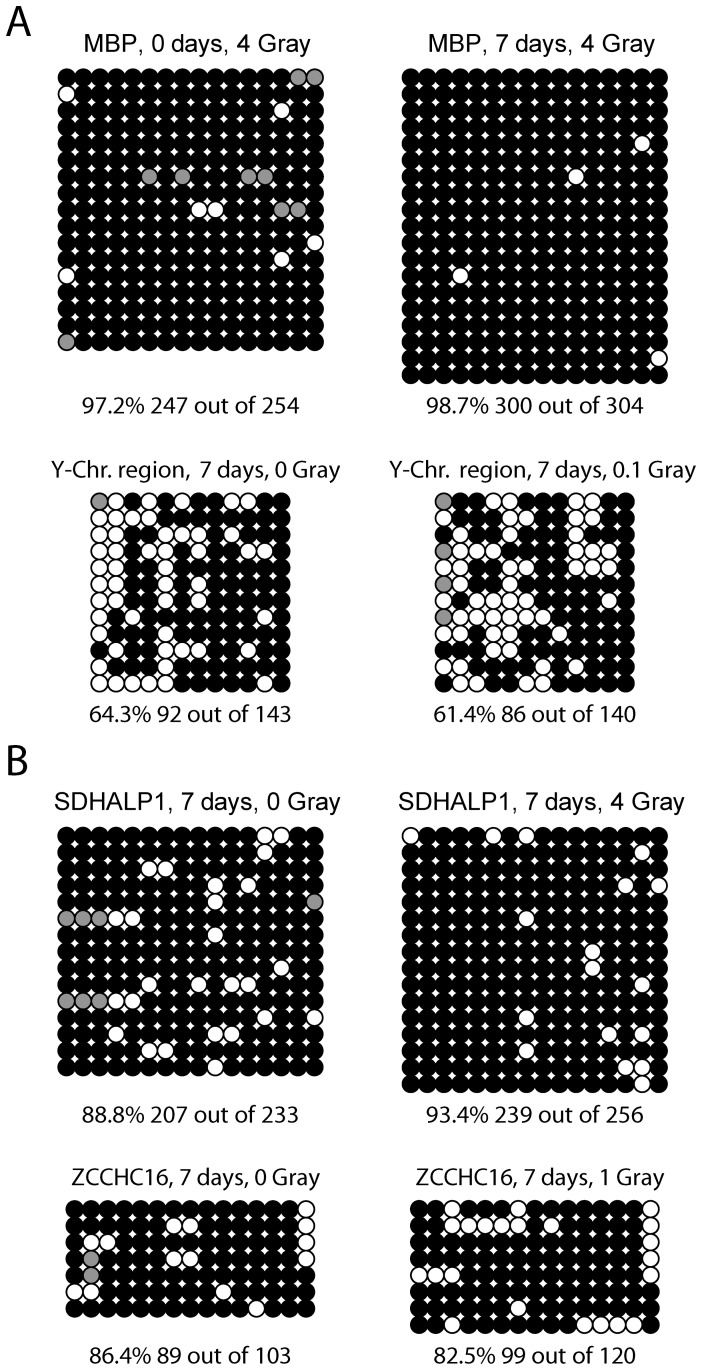
Bisulfite sequencing. The Figure shows the result of the bisulfite sequencing analysis. Each circle represents one CpG in the PCR product. The black circles indicate a methylated CpG and white circles indicate an unmethylated CpG. The gray circles show CpGs with an uncertain methylation status. Each row represents a single clone. **A.** The gene *MBP* and a region of the Y chromosome are shown. **B.** The genes *SDHALP1* and *ZCCHC16* are shown. On the left side, the data for untreated (control) cells are shown. The panels on the right show data for the irradiated cells. The bottom line describes the total fraction of methylated CpGs for each treatment and control.

## Results

We used a ^137^Cs ionizing radiation source to irradiate primary human diploid fibroblasts and normal human bronchial epithelial cells. The goal of these experiments was to determine if ionizing radiation is able to induce changes in DNA cytosine methylation patterns. The radiation doses were chosen to be well tolerated by the cells with no signs of cell death immediately following irradiation or up to seven days after irradiation as assessed by trypan blue dye exclusion. Fibroblasts and bronchial epithelial cells did not undergo further cell division within seven days after being exposed to radiation doses of 4 Gy or higher (Figure S1). Normal bronchial cells were exposed to doses of up to 4 Gy and fibroblasts were exposed up to a maximum of 10 Gy.

We treated NHBECs and HFBs with different doses of γ-radiation and let them recover for 7 days to allow potential aberrations in DNA methylation patterns to manifest themselves. We used the MIRA method (Figure 1) to score genome-wide changes of the DNA methylation patterns after γ-radiation. The microarray platforms used were Nimblegen CpG island plus promoter arrays. These arrays contain all defined CpG islands of the human genome (n = 27,728) and all Refseq gene annotated promoters (n = 22,532). Using bioinformatics analysis, we identified and catalogued all significant methylation peaks in control and irradiated cells (see Materials and Methods). Peak differences, i.e. between irradiated and control cells, were based on a difference factor of 2-fold or greater. Using this analysis, we identified a number of apparent peak differences on the microarrays. The highest number of differential peaks occurred in NHBEC in the comparison between the 4 Gy treatment with immediate harvest and the 4 Gy treatment and 7 days recovery time with 224 hypomethylated and 218 hypermethylated peaks (Table 1). In the HFBs, the highest number of peak differences occurred in the comparison between the 0 Gy treatment and 7 days recovery time and 1 Gy and 7 days recovery time after the treatment with 60 hypermethylated and 118 hypomethylated peaks (Table 1). The numbers of differential methylation peaks were relatively small, in particular for the irradiated fibroblasts, and in most cases dropped even further when Loess normalization was used to correct dye intensity bias (Table 1). Importantly, the fold-difference ratios were rarely greater than 3-fold (Table 1; the highest differential peak identified had a difference of 3.5-fold). When analyzing the data using Nimblegen’s SignalMap software, most areas along the chromosomes did not show any substantial differences. Examples are shown in Figure 2.

After identifying several apparent peak differences between controls and γ-radiation treatments based on bioinformatics analysis (Table 1; Figure 3), we used the methylation assay COBRA (combined bisulfite restriction analysis) to confirm the most pronounced differences. For NHBEC, we selected the genes *MBP*, *CLEC18C* and a gene-less region of the Y chromosome (Figure 3A). For the human fibroblasts, we selected the genes *CLEC18A*, *SDHALP1* and *ZCCHC16*, which showed appreciable peak differences by microarray analysis (Figure 3B). After performing bisulfite treatment of the DNA, PCR amplification and restriction digestion with enzymes having CpG-containing recognition sites, we found no changes in the epigenetic patterns for all analyzed genes and irradiation treatments (Figure 4).

To confirm these results further, we performed a bisulfite (BS) sequencing analysis of the same samples (Figure 5). Although the microarray analysis indicated hypomethylation at this position, bisulfite sequencing of the *MBP* gene showed a marginal increase of methylation of about 1.5% (percent of all analyzed CpGs methylated) after treatment with 4 Gy of radiation and a recovery time of 7 days in the NHBECs. In another bisulfite-based sequence analysis, the methylation status of the gene-less region on the Y chromosome decreased by only about 2.95% after the treatment with 0.1 Gy and 7 days recovery time in the NHBECs (Figure 5A).

A similar result was obtained for the HFBs. The methylation status of the *SDHAP1* gene increased after treatment with 4 Gy and 7 days recovery time by about 4.6%. In contrast to that, the methylation status decreased by about 3.9% in the gene *ZCCHC16* after treatment with 1 Gy of ionizing radiation and 7 days of recovery time. Both genes had shown some degree of hypomethylation by microarray analysis (Figure 3B). Overall, the candidate differential peaks scored on the microarrays (a relatively small number of peaks with low –fold difference ratios) could not be confirmed by independent methylation assays and therefore must be considered false positives of the array analysis. Such a small number of false positives can be expected when scoring relatively small differences for a total number of tens of thousands of CpG islands and promoters encompassing the entire human genome.

## Discussion

Radioactive pollution and nuclear bomb threats are very serious problems for the environment and for human health and survival. It is well understood in which way ionizing radiation damages DNA, causes mutations and DNA strand breakage in animals and plants [14], [15], [16], [17] and causes cancer in humans [18], [19], [20], [21], [22], [23], [24], [25], [26], [27]. However, almost nothing is known how this type of radiation may affect the epigenome.

Only a few publications describe research on the epigenetic influence of ionizing radiation [28]. In most of these earlier studies, only global cytosine methylation levels were analyzed. O’Hagan et al induced a defined double-strand break using a nuclease and observed recruitment of chromatin silencing factors including DNMT3B resulting in occasional heritable silencing of the locus [29]. However, a genome-wide study of specific genes following ionizing radiation is lacking. In our study, we investigated the epigenetic effects of γ-radiation on two normal human cell types, fibroblasts and bronchial epithelial cells. After different exposures to γ-radiation, we analyzed the effects on the epigenome via the specific and sensitive MIRA method based on microarray analysis (Figures 2 and 3), which characterizes DNA methylation changes. We tried to confirm the highest microarray peak differences (e.g., Figure 3) via COBRA (Figure 4) and bisulfite sequencing (Figure 5). These apparent peak differences could not be confirmed by these methods, but both were consistent with each other and showed the same result. Therefore, the microarray results represent false positives. The peak differences scored were never more than 3.5-fold and rarely more than 3-fold. This is in sharp contrast to our previous studies using the same approach to analyze differences in DNA methylation between normal and tumor tissues. In these studies, much higher fold- difference ratios, in the order of 5-15-fold, are routinely observed for hundreds if not thousands of gene loci, and can readily be confirmed by bisulfite-based analyses [30], [31]. Our conclusion is that γ-radiation has no appreciable influence on DNA methylation patterns in the context of our experimental system. If there were any differences, they would be occurring at a very small scale (perhaps only a few differences with minor or partial changes in methylated CpG density at specific loci) and/or in only a small fraction of the cell population and could not be picked up by the MIRA microarray approach. Therefore, ionizing radiation does not induce a general mechanistic pathway, for example by initiating a signaling cascade that would lead to generalized changes in DNA methylation patterns.

Even with a high level of radiation (10 Gy), an epigenetic shift at the level of DNA methylation did not appear. Also, after an exposure at this level, an epigenetic alteration would be biologically irrelevant because in humans a whole body exposure with 6 to 10 Gy is with and without supportive care almost 100% lethal. Even an exposure to 3.5 Gy leads to a mortality of 50% after 60 days (*LD*_50/60_) without any healthcare [32].

We cannot, however, exclude the possibility that ionizing radiation could alter DNA methylation patterns after a long-lasting chronic exposure at much lower doses. Since ionizing radiation is known to alter gene expression patterns and induces a large number of DNA damage response genes [33], it may alter other transcription-associated chromatin marks, including histone modifications, perhaps resulting in a transient or even permanent change to the chromatin and epigenome upon prolonged irradiation that is independent of DNA methylation. However, DNA methylation is considered to be the most stable epigenetic modification. Our data suggest that ionizing radiation does not alter this stable epigenetic mark directly upon exposure.

## Materials and Methods

### Cells and Biological Materials

Normal human diploid fibroblasts and normal human bronchial epithelial cells (Lonza; Anaheim, CA) were grown in DMEM and BEGM™ (Bronchial Epithelial Cell Growth Medium BulletKit™; Lonza) medium, respectively. The restriction enzymes for the COBRA analysis, Taq^α^I (5′-TCGA-3′), BstUI (5′-CGCG-3′) and HpyCH4IV (5′-ACGT-3′), were obtained from New England Biolabs (Ipswich, MA).

### Ionizing Radiation Treatment

As a radiation source, we used the synthetic radioactive nuclide Cesium 137 (^137^Cs) from an irradiator (J.L. Shepherd and Associates). Both types of normal human cells were grown in T75 flasks. The fibroblast cells were treated with doses of 0.1, 1, 4, and 10 Gray. After an immediate change of the cell culture medium, the cells were given a recovery period of 7 days followed by cell harvesting. Two controls were included, one in which the cells were treated with 10 Gray of radiation followed by immediate harvesting and one in which the cells were harvested after 7 days of growth without any irradiation. The NHBEC cells were treated with 0.1, 1, and 4 Gray. After an immediate medium change, a recovery time of 7 days was allowed before harvesting. Controls included no radiation treatment and a treatment with 4 Gray and immediate harvesting.

### DNA Isolation

After the specific γ-radiation treatment, the cells were trypsinized and pelletted. Following a proteinase K treatment, DNA was isolated with a standard phenol/chloroform method and by ethanol precipitation.

### MIRA and Microarray Analysis

To detect potential genome-wide changes in DNA methylation patterns after the γ radiation treatment, the methylated-CpG island recovery assay (MIRA) combined with microarray analysis was used [34], [35], [36]. Nimblegen’s Signalmap program was used to visualize the DNA methylation data (http://www.nimblegen.com/products/software/signalmap/index.html).

### Bioinformatics Analysis

All the data were quantile-normalized first before analysis. In one set of comparisons, Loess normalization was used to correct intensity-dependent dye bias. Probes were considered positive if their normalized log2 ratio were above 2-fold. Peaks in each sample were defined as four or more consecutive positive probes with either one or no gaps. To identify hypermethylated peaks in treated samples vs. untreated samples, the average probe log2 ratio signals within the peaks identified in each treatment sample were compared to the untreated sample. Only the peaks with an average log2 ratio signal difference of more than 1 (2-fold) were considered hypermethylated peaks. Hypomethylated peaks were the peaks identified in the untreated sample and having an average log2 ratio signal difference of more than 1 compared to the treated sample. These peaks were annotated to the Refseq transcript database downloaded from UCSC genome database. Microarray data have been deposited into the GEO database (accession number GSE39038).

### DNA Methylation Analysis by COBRA

For confirmation of the analyzed MIRA signals indicating a potential loss or gain of DNA methylation, the candidate locus was investigated by combined bisulfite restriction analysis (COBRA) [37]. PCR was performed with primers and conditions listed in Table S1 and Table S2. Briefly, COBRA-PCR was performed with bisulfite DNA-specific primers using 50 ng of bisulfite modified genomic DNA as template for 60 cycles after a 15 min incubation at 95°C, then 30 sec at the T_A_ (see Table S2) and 30 sec at 72°C in 25 µl containing 5 nmol dNTPs, 20 pmoles of primers, and 1.25 units of Hot start *Taq* DNA polymerase (Qiagen, Valencia, CA). Five microliters of the PCR product was analyzed on a 2% Tris–borate-EDTA agarose gel. Equal amounts of PCR product were digested with the adequate restriction enzyme (see Table S2), Taq^α^I (5′-TCGA-3′), BstUI (5′-CGCG-3′) or HpyCH4IV (5′-ACGT-3′).

### Bisulfite Sequencing

A COBRA PCR was performed as described above. The PCR product was ligated into a cloning vector (TOPO® Cloning Kit; Invitrogen, Grand Island, NY, or the pGEM®-T-Easy Kit, Promega, Madison, WI) and transformed into competent cells. Different clones were picked at random, the plasmid isolated and sequenced. For the analysis of methylated and unmethylated cytosines, the free software program Bioedit was used (http://www.mbio.ncsu.edu/bioedit/bioedit.html).

## Supporting Information

Figure S1 **Growth curves of cells after exposure to different doses of ionizing radiation.** Cells were irradiated with the indicated doses of ionizing radiation and cell numbers were determined after three days and seven days. The experiments were carried out in quadruplicates (mean +/− S.D.). (A) Human fibroblasts; (B) human bronchial epithelial cells.
(TIF)

Table S1 **Oligonucleotide primers.**
(DOCX)

Table S2 **PCR parameters.**
(DOCX)
