# Supplementary figures and images for: Gamma Irradiation Does Not Induce Detectable Changes in DNA Methylation Directly following Exposure of Human Cells
Source: PLoS One. 2012 Sep 14;7(9):e44858. doi: 10.1371/journal.pone.0044858 (PMC3443085; doi:10.1371/journal.pone.0044858)

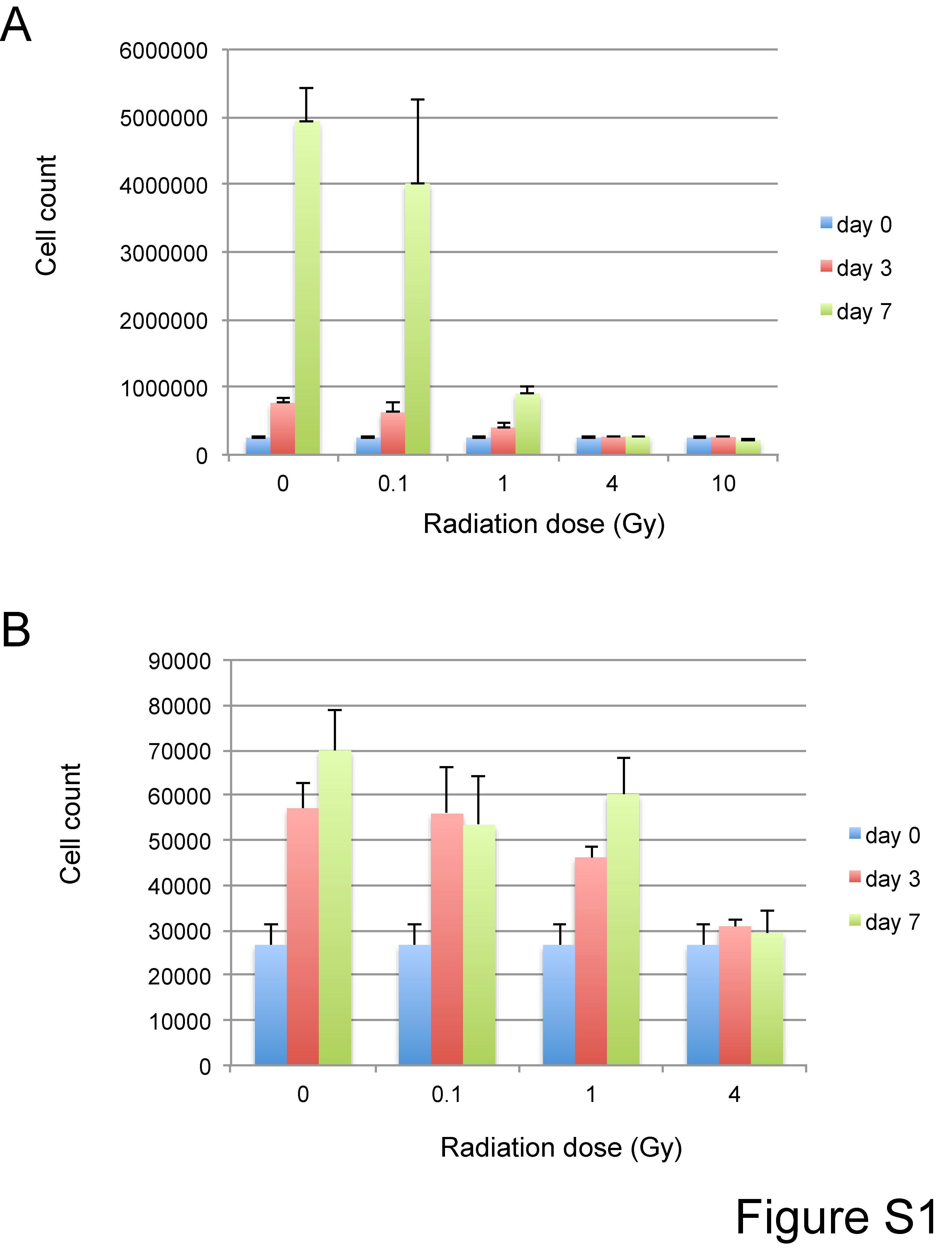

Supplement: Figure S1 — Growth curves of cells after exposure to different doses of ionizing radiation. Cells were irradiated with the indicated doses of ionizing radiation and cell numbers were determined after three days and seven days. The experiments were carried out in quadruplicates (mean +/− S.D.). (A) Human fibroblasts; (B) human bronchial epithelial cells. (TIF) [file pone.0044858.s001.tif]
