# Supplementary material for: Gamma Irradiation Does Not Induce Detectable Changes in DNA Methylation Directly following Exposure of Human Cells
Source: PLoS One. 2012 Sep 14;7(9):e44858. doi: 10.1371/journal.pone.0044858 (PMC3443085; doi:10.1371/journal.pone.0044858)
Supplement: Table S1 — Oligonucleotide primers. (DOCX) [file pone.0044858.s002.docx]

**Table S1: Oligonucleotide primers**

| **Cell type** | **Gene** | **Upper Primer (5’ 🡪 3’)** | **Lower Primer (5’ 🡪 3’)** |
| --- | --- | --- | --- |
|  |  | Oligonucleotides for *combined bisulfite restriction-analysis* (COBRA) | |
| HFB  (human fibroblasts) | *CLEC18A* | 5’GAA TTT TTT TGG GGT TTT GTT TAG T | 5’AAT CAA TAT ATC CAA ACA ACC TCC TAT TA |
|  | *SPEG* | 5’TTT TAG AGG AGT AAG TGA GTT GGT G | 5’AAA ACA ATT CA ACC CAA ATA AAA C |
|  | *SDHALP1* | 5’TTT GTA TGT ATT GAA GTT ATT GTT TGT | 5’ATT TAA ACC TCA TTC CTC ATT ACC A |
|  | *ASB10* | 5’TTT AGT TAA TGG GGT GAG GTG | 5’CAA CCT CAA AAC CTA CCC TTA C |
|  | *ZCCHC16* | 5’TTT TTA TGA TGA TGT TTT GGG TTG | 5’CAA AAA ACA AAA ACC CTC CC |
| NHBEC  (normal human bronchial epithelial cells) | *MBP* | 5’TTT GTT GTG GTT AGG TAT TTG GAT T | 5’CCC CTC AAA AAA AAC TAC TTT ATC C |
|  | *CLEC18C* | 5’TTG AGA AAT TTG GTT TAT GGT GTA G | 5’AAA AAT ACA ATC TAT AAA CTC AAA AAA AA |
|  | *ZNF187* | 5’GTA GTT TAA AGG GTT GTA GGG T | 5’TCT TTT CCT TAA TTA AAC ACT TCC C |
|  | *MAGED1* | 5’GTA AGG GGT GGG ATT AAG AGG TAT A | 5’CAC TCT CTC CTT ATC CAA AAA AAA |
|  | *SLC38A2* | 5’TTT TTA ATG TTT TAT TGA GGA AAT T | 5’AAC AAA AAT AAC TAT TAT TTA AAA CTA |
|  | *Y chr. region* | 5’TTT TGT TTA GGA TTG TTT AGA TTT | 5’TTA TCA TTA CCT TTA CAA AAA TTC C |
|  | *SFT2D3* | 5’GTT GAG GTT TTA GGA GGA GGA ATA G | 5’AAA AAA ATC ATT AAC AAA ACC CAC TC |
