# Supplementary material for: Gamma Irradiation Does Not Induce Detectable Changes in DNA Methylation Directly following Exposure of Human Cells
Source: PLoS One. 2012 Sep 14;7(9):e44858. doi: 10.1371/journal.pone.0044858 (PMC3443085; doi:10.1371/journal.pone.0044858)
Supplement: Table S2 — PCR parameters. (DOCX) [file pone.0044858.s003.docx]

**Table S2: PCR parameters**

| **Cell type** | **Gene** | **Length in**  **base pairs** | **Annealing**  **temperature (T_A_)** | **PCR**  **cycles** | **Restriction**  **enzyme** | **Formamide**  **concentration** |
| --- | --- | --- | --- | --- | --- | --- |
|  |  | PCR parameters for combined bisulfite restriction-analysis (COBRA) | | | | |
| HFB  (human fibroblasts) | *CLEC18A* | 370 | 52°C | 60 | TaqαI | 2% |
|  | *SPEG* | 312 | 52°C | 60 | TaqαI | 2% |
|  | *SDHALP1* | 390 | 52°C | 60 | TaqαI | 2% |
|  | *ASB10* | 274 | 60°C | 60 | BstUI | 2% |
|  | *ZCCHC16* | 228 | 55°C | 60 | BstUI | 2% |
| NHBEC  (normal human bronchial epithelial cells) | *MBP* | 394 | 52°C | 60 | HpyCH4IV | 2% |
|  | *CLEC18C* | 368 | 52°C | 60 | HpyCH4IV | 2% |
|  | *ZNF187* | 291 | 52°C | 60 | HpyCH4IV | 2% |
|  | *MAGED1* | 318 | 52°C | 60 | HpyCH4IV | 2% |
|  | *SLC38A2* | 230 | 50°C | 60 | TaqαI | 2% |
|  | *Y chr. rgion* | 219 | 50°C | 60 | TaqαI | 2% |
|  | *SFT2D3* | 246 | 55°C | 60 | TaqαI | 2% |
